# Simple tandem repeat (TTTA)_n _polymorphism in CYP19 (aromatase) gene and breast cancer risk in Nigerian women

**DOI:** 10.1186/1477-3163-5-12

**Published:** 2006-05-09

**Authors:** Michael N Okobia, Clareann H Bunker, Joseph M Zmuda, Emmanuel R Ezeome, Stanley NC Anyanwu, Emmanuel EO Uche, Joseph Ojukwu, Lewis H Kuller, Robert E Ferrell

**Affiliations:** 1 Department of Epidemiology, Graduate School of Public Health, University of Pittsburgh, Pittsburgh, PA 15261, USA; 2 Department of Human Genetics, Graduate School of Public Health, University of Pittsburgh, Pittsburgh, PA 15261, USA; 3 Department of Surgery, University of Benin Teaching Hospital, Benin City, Nigeria; 4 Department of Surgery, University of Nigeria Teaching Hospital, Enugu, Nigeria; 5 Department of Surgery, Nnamdi Azikiwe University Teaching Hospital, Nnewi, Nigeria; 6 Department of Surgery, University of Port Harcourt Teaching Hospital, Port Harcourt, Nigeria

## Abstract

**Background:**

Breast cancer is the most common cancer and the leading cause of cancer related deaths in women worldwide. The incidence of the disease is increasing globally and this increase is occurring at a faster rate in population groups that hirtherto enjoyed low incidence. This study was designed to evaluate the role of a simple tandem repeat polymorphism (STRP) in the aromatase (CYP19) gene in breast cancer susceptibility in Nigerian women, a population of indigenous sub-Saharan African ancestry.

**Methods:**

A case-control study recruiting 250 women with breast cancer and 250 women without the disease from four University Teaching Hospitals in Southern Nigeria was carried out between September 2002 and April 2004. Participants were recruited from the surgical outpatient clinics and surgical wards of the Nigerian institutions. A polymerase chain reaction (PCR)-based assay was employed for genotyping and product sizes were detected with an ABI 3730 DNA Analyzer.

**Results:**

Conditional logistic regression analysis revealed that harboring the putative high risk genotypes conferred a 29% increased risk of breast cancer when all women in the study were considered (Odds ratio [OR] = 1.29, 95% confidence interval [CI] 0.83–2.00), although this association was not statistically significant. Subgroup analysis based on menopausal status showed similar results among premenopausal women (OR = 1.35, 95% CI 0.76–2.41 and postmenopausal women (OR = 1.27, 95% CI 0.64–2.49). The data also demonstrated marked differences in the distribution of (TTTA)_n _repeats in Nigerian women compared with other populations.

**Conclusion:**

This study has shown that harboring 10 or more repeats of the microsatellite (TTTA)_n _repeats of the CYY19 gene is associated with a modest increased risk of breast cancer in Nigerian women.

## Background

An aging world population with rising global burden of breast cancer [1], emerging evidence suggesting that postmenopausal obesity as well as weight gain over the adult years are positively associated with postmenopausal breast cancer [2] and the finding that breast cancer patients may have an inherently higher aromatase expression in breast adipose tissue when compared to healthy women [3] have increased research interest on mechanisms underlying aromatase action in breast carcinogenesis.

The CYP19 gene encodes aromatase, which is responsible for the rate-limiting step in the metabolism of C19 steroids to estrogens and is expressed in most breast carcinomas [4]. Five different polymorphisms in the CYP19 gene have been described in relationship to breast cancer risk, including the highly polymorphic tetranucleotide repeats in intron 5 [5-9], a C-T substitution in the 3' noncoding region of exon 10 [10], a cytosine to thymine substitution in codon 264 of exon 7 (resulting in amino acid Arginine to Cysteine substitution) [11], a silent G-A polymorphism at codon 80 in exon 3 [6], and a rare 3 base pair change within the promoter region of exon 1.4 [9]. Although the tetranucleotide repeat polymorphism is the most widely studied of all these variants in the CYP19 gene, there are a lot of inconsistencies in reports in various populations with respect to breast cancer susceptibility [5-9]. There is evidence suggesting that part of the population differences in breast cancer risk may be related to the wide racial variation in the distribution of various polymorphisms in different populations. This study was designed to evaluate the role of the tetranucleotide repeat polymorphism in breast cancer risk in an indigenous African population in Southern Nigeria.

## Patients and methods

### Study subjects

A total of 500 study participants comprising 250 women with breast cancer and 250 age-matched control women without the disease were recruited from four University Teaching Hospitals located in Southeastern and Midwestern Nigeria, including University of Benin Teaching Hospital, Benin City; University of Nigeria Teaching Hospital, Enugu; Nnamdi Azikiwe University Teaching Hospital, Nnewi; and University of Port Harcourt Teaching Hospital, Port Harcourt. Recruitment was carried out between September 2002 and April, 2004. The study protocol was approved by the Ethics and Research Committees of the participating Nigerian Institutions and the Institutional Review Board of the University of Pittsburgh.

Written informed consent was obtained from participants willing to participate in the study prior to commencement of recruitment. At the end of the questionnaire session, anthropometric measurements including height, weight, waist and hip circumferences were taken.

### Biological samples

Biological samples including 40 milliliters of blood in two 15-milliliter plain vacutainer tubes and one 10 milliliter K_3_-EDTA tube were collected at the end of the questionnaire session. Samples were centrifuged within 10 h of collection; buffy coat was separated from plasma and red cells in the K_3_-EDTA vacutainer tubes and carefully aliquoted into 3 ml tubes while clots separated from serum in the plain vacutainer tubes were turned into two 20 ml plain tubes and stored at -20°C in each of the Nigerian study sites until transferred in ice packs to the Nigerian coordinating center at the University of Benin Teaching Hospital where samples were stored at -20°C until transferred to the University of Pittsburgh in dry ice packs. Samples were then stored at -80°C at the University of Pittsburgh until DNA extraction.

### DNA extraction

DNA was extracted from buffy coats using the QIAamp Mini Kit protocol (Qiagen, Chatsworth, CA) or blood clots using the QIAamp Midi Kit protocol (Qiagen, Chatsworth, CA) for participants in whom buffy coat was unavailable. The DNA was stored at 4°C until amplified by polymerase chain reaction (PCR) and used for genotyping on the ABI 3730 DNA Analyzer (Applied Biosystems, Foster City, CA).

### PCR and genotyping

PCR amplification of the polymorphic fragment was generated using primers, 5'-CAA CTC GAC CCT TCT TTA TG-3' (forward) and 5'-GTT TGA CTC CGT GTG TTT GA-3' (reverse). The forward primer was 5'-labeled with a fluorescent dye (FAM, 5-carboxy-flouorescein) for automated fragment analysis. A 50 μl reaction mixture containing 2 μl of genomic DNA, 8 μl of deoxynuclotide triphosphates, 0.5 μl each of forward and reverse primers, 5 μl of 10× buffer, 1.5 μl of MgCl and 2 μl of Taq polymerase was placed in Thermalcycler (MJ Dyad PCR machine). After denaturing for 5 min at 95°C, the DNA was amplified for 35 cycles at 95°C for 30 s, 55°C for 30 s, 72°C for 45 s, followed by a 5 min extension at 72°C. A positive control containing genomic DNA and a negative control containing everything except DNA were included in the PCR experiment. Five μl of each PCR product, including the controls, were run on a 1% agarose gel to ensure that the expected fragment size was generated.

The product sizes were then detected with an ABI 3730 DNA Analyzer (Applied Biosystems, Foster City, CA) at the Genomics and Proteomics Core Laboratory at the University of Pittsburgh. Allele calling was carried out using Genotyper software (Applied Biosystems, Foster City, CA).

### Statistical analysis

Data analysis was carried out using Statistical Analysis Software (SAS), Version 8.0. Association between demographic, reproductive and anthropometric variables and breast cancer risk was first assessed using conditional logistic regression. Each matched case was paired with the corresponding control to enable differences between the cases and controls to be calculated. First each variable was assessed alone in a univariate model. The strength of significant variables was further assessed by building multivariate models.

Conditional logistic regression was also used to assess the relationship between the distribution of the genotypes of the CYP19 tetranucleotide (TTTA)_n _repeats and breast cancer risk. Based on information available in the literature, the CYP19 genotypes were categorized into two groups for the purpose of conditional logistic regression. There is evidence suggesting that an increased risk of breast cancer is associated with 10 or more (TTTA)_n _repeats. Genotypes containing at least one allele with 10 or more repeats were categorized as high risk genotypes while genotypes containing alleles with less than 10 repeats were categorized as low risk genotypes. Because of the small number of individuals carrying these genotypes, we had to collapse all genotypes in the high risk category as one group in the logistic regression model. A multivariate conditional logistic regression model containing the significant predictors of breast cancer risk in the descriptive analysis and the CYP19 genotype data was then developed to assess gene environment interactions.

## Results

### Association of demographic/reproductive characteristics with breast cancer risk

Conditional logistic regression analysis was used to examine the association between demographic/reproductive variables of study participants and breast cancer risk. In the final multiple conditional logistic regression model, only family history of breast cancer (OR = 8.07, 95% CI 1.003–64.95), waist/hip ratio (WHR) (OR = 1.98, 95% CI 1.27–3.10), age at first full term pregnancy (OR = 1.31, 95% CI 1.01–1.71), and higher level of education (OR = 1.33, 95% CI 1.05–1.74) were found to be significant predictors of breast cancer risk.

### Distribution of CYP19 (TTTA)_n _repeat alleles

#### All women

Seven different alleles (designated A1 to A7, as shown in Table 1) of the tetranucleotide (TTTA)_n _repeat polymorphism of the CYP19 gene were identified in 228 cases and 228 control subjects in whom polymerase chain reaction (PCR) amplification and genotyping were successful. The most common alleles were A1 (356 base pairs [bp], 7 repeats) and A2 (360 bp, 8 repeats) alleles. The A1 allele was slightly more common in the cases (allele frequency 0.3385) than in the control subjects (allele frequency 0.3026) while allele A2 was less common in the cases (allele frequency 0.4978) compared to the control subjects (allele frequency 0.5504) as shown in Table 1. The distribution of these alleles was not significantly modified by menopausal status.

**Table 1:** Allele distribution of the polymorphic CYP19 in breast cancer cases and controls: number of alleles (allele frequency)

Allele type	Cases	Controls
	Number (Frequency)	Number (Frequency)
A1 (356) 7 repeats	152 (0.3385)	144 (0.3026)
A2 (360) 8 repeats	228 (0.4978)	251 (0.5504)
A3 (364) 9 repeats	13 (0.0310)	15 (0.0329)
A4 (368) 11 repeats	2 (0.0088)	0 (0.0000)
A5 (376) 12 repeats	38 (0.0819)	31 (0.0724)
A6 (380) 13 repeats	19 (0.0240)	17 (0.0395)
A7 (384) 14 repeats	0 (0.0000)	1 (0.0022)

### Distribution of CYP19 (TTTA)_n _repeat polymorphism genotypes

#### All women

The distribution of the various genotypes of the CYP19 (TTTA)_n _repeat polymorphism are shown in Table 2. Homozygosity for the A1 (7 repeats) allele was more common in the cases (27 [11.84%]) than in the control subjects (19 [8.33%]) while homozygosity for the A2 (8 repeats) allele was less common in the cases (59 [25.89%]) compared to the control subjects (65 [28.51%). Heterozygosity for the A1 and A2 alleles (A1A2 genotype) was less common in the cases (73 [32.02%]) compared to the control subjects (83 [36.40%]). Other less common genotypes are as shown in Table 2.

**Table 2:** (TTTA)_n _repeat polymorphism genotype in breast cancer cases and controls (All women)

Genotype	Cases		Controls	
	Number (n)	Percentage (%)	Number (n)	Percentage (%)
A1A1 (356/356)	27	11.84	19	8.33
A1A2 (356/360)	73	32.02	83	36.40
A1A3 (356/364)	7	3.07	6	2.63
A1A4 (356/368)	1	0.44	0	0.00
A1A5 (356/376)	11	4.82	9	3.95
A1A6 (356/380)	6	2.63	4	1.75
A2A2 (360/360)	59	25.89	65	28.51
A2A3 (360/364)	4	1.75	7	3.07
A2A4 (360/368)	1	0.44	0	0.00
A2A5 (360/376)	21	9.21	18	7.89
A2A6 (360/380)	12	5.26	11	4.82
A2A7 (360/384)	0	0.00	1	0.44
A3A3 (364/364)	1	0.44	1	0.44
A3A5 (364/376)	1	0.44	0	0.00
A4A4 (368/368)	1	0.44	0	0.00
A5A5 (376/376)	3	1.32	2	0.88
A5A6 (376/380)	0	0.00	2	0.88
Total	228	100.00	228	100.00

**Risk associated with at least one allele containing 10 or more repeats**: OR = 1.29, 95% CI 0.83–2.00

**Risk associated with homozygosity for alleles containing 10 or more repeats**: OR = 4.00, 95% CI 0.45–35.79

#### Premenopausal women

The distribution of the CYP19 (TTTA) genotypes in premenopausal women is similar to the general pattern seen in all women. The most common genotypes include A1A2 (44 [34.92%] in cases, 50 [38.76%] in controls), and A2A2 (31 [24.60%] in cases, 36 [27.91%] in controls) as shown in Table 3.

**Table 3:** (TTTA)_n _repeat polymorphism genotype in breast cancer cases and controls (Premenopausal women)

Genotype	Cases		Controls	
	Number (n)	Percentage (%)	Number (n)	Percentage (%)
A1A1 (356/356)	17	13.49	9	6.98
A1A2 (356/360)	44	34.92	50	38.76
A1A3 (356/364)	2	1.59	3	2.33
A1A4 (356/368)	1	0.79	0	0.00
A1A5 (356/376)	8	6.35	5	3.88
A1A6 (356/380)	2	1.59	5	3.88
A2A2 (360/360)	31	24.60	36	27.91
A2A3 (360/364)	2	1.59	5	3.88
A2A4 (360/368)	1	0.79	0	0.00
A2A5 (360/376)	11	8.73	10	7.75
A2A6 (360/380)	5	3.97	5	3.88
A4A4 (368/368)	1	0.79	0	0.00
A5A5 (376/376)	1	0.79	1	0.79

Totals	126	100.00	129	100.00

**Risk associated with at least one allele containing 10 or more repeats**: OR = 1.35, 95% CI 0.76–2.41

#### Postmenopausal women

Of 108 postmenopausal breast cancer cases and 108 age-matched control subjects in the study, PCR amplification and genotyping was successful in 102 cases and 99 controls. As shown in Table 4, the A1A2 genotype was slightly less common in the cases (29 [28.43%] compared to the control subjects (33 [33.33%]) while the A2A2 genotype was almost equally distributed among the cases (28 [27.45%]) and controls (29 [29.29%]).

**Table 4:** (TTTA)_n _repeat polymorphism genotype in breast cancer cases and controls (Postmenopausal women)

Genotype	Cases		Controls	
	Number (n)	Percentage (%)	Number (n)	Percentage (%)
A1A1 (356/356)	10	9.90	10	10.10
A1A2 (356/360)	29	28.43	33	33.33
A1A3 (356/364)	5	4.90	3	3.03
A1A5 (356/376)	3	2.94	4	4.04
A1A6 (356/380)	4	3.92	0	0.00
A2A2 (360/360)	28	27.45	29	29.29
A2A3 (360/364)	2	1.96	2	2.02
A2A5 (360/376)	10	9.80	7	7.07
A2A6 (360/380)	7	6.86	6	6.06
A2A7 (360/384)	0	0.00	1	1.01
A3A3 (364/364)	1	0.98	1	1.01
A3A5 (364/376)	1	0.98	0	0.00
A5A5 (376/376)	2	1.96	1	1.01
A5A6 (376/380)	0	0.00	2	2.02

Totals	102	100.00	99	100.00

**Risk associated with at least one allele containing 10 or more repeats**: OR = 1.27, 95% CI 0.64–2.49

**Risk associated with homozygosity for alleles containing 10 or more repeats**: OR = 2.00, 95% CI 0.18–22.05

### Association of CYP19 (TTTA)_n _genotypes and breast cancer risk

Conditional logistic regression was used to evaluate the association between the distribution of the CYP19 (TTTA)_n _genotypes and breast cancer risk. For the purpose of this analysis, the genotypes were categorized into two broad groups; genotypes with at least one allele containing 10 or more (TTTA)_n _repeats were classified as high-risk genotypes while those containing less than ten alleles were considered as low-risk genotypes. This classification was adopted based on evidence in the literature suggesting that the higher number of repeats (10 and 12 repeats) were associated with increased risk of breast cancer. Conditional logistic regression analysis showed a 29% increased risk of breast cancer for harboring at least one high risk allele [[10] or more (TTTA)_n _repeats] when all women were considered (0R = 1.29, 95% CI 0.83–2.00). There was a 35% increased risk of premenopausal breast cancer for women harboring at least one high risk allele (OR = 1.35, 95% CI 0.76–2.41). Harboring the high risk genotype was associated with a 27% increased risk of postmenopausal breast cancer (0R = 1.27, 95% CI 0.64–2.49).

The risk profile of women homozygous for any of the high risk alleles was further considered. Four breast cancer cases were homozygous for any of the high risk alleles; three of these women carried the homozygous A5A5 genotype while one was homozygous for the A4A4 [[10] (TTTA)_n _repeats]. Two control participants were homozygous for any of the high risk alleles. When all women were considered together, there was a 4-fold increased risk of breast cancer for women carrying the homozygous genotypes of any of the high risk alleles although this was not statistically significant (OR = 4.00, 95% CI 0.45–35.79). There was a 2-fold increased risk of postmenopausal breast cancer (OR = 2.00, 95% CI 0.18–22.05) for carriers of the homozygous genotypes.

## Discussion

This study has demonstrated marked variation in the distribution of the tetranucleotide (TTTA)_n _repeats of the CYP19 (aromatase) gene among Nigerian women compared with other population groups. The (TTTA)_n _repeats ranged from seven to 14 repeats with seven and eight repeats constituting the major alleles with allele frequencies of 0.3385 and 0.3026 for the seven repeat allele in the cases and controls respectively and 0.4978 and 0.5504 for the eight repeat allele in cases and controls respectively. Among some Caucasian population groups, the seven repeat allele appears to be the most common, with allele frequency of 0.484 in the Nordic population [5] and 0.50 in non-Latina Whites in Southern California [9]. In the British population, the six repeat allele appears to be the most common (allele frequency of 0.379) [8] with the seven repeat allele frequency of 0.021. A much higher frequency of 0.76 was reported for the seven repeat allele among African American women in southern California [9]. Populations of Japanese descent appear to harbor the highest frequency of the seven repeat allele (allele frequency of 0.85) among Japanese Americans in Southern California and Hawaii [9].

Our data suggest that harboring at least one 10 or more repeats was associated with a 29% increased risk of breast cancer (OR = 1.29, 95% CI 0.83–2.00) and this risk profile was unaltered in premenopausal (OR = 1.35, 95% CI 0.76–2.41) and postmenopausal women (OR = 1.27, 95% CI 0.64–2.49). It is important to note that four of the six study participants that harbored the homozygous variants of the putative high risk alleles were cases. Although not statistically significant, probably due to the small number of cases with these homozygous genotypes, there was a 4-fold increased risk of breast cancer in women who were homozygous for these alleles (OR = 4.00, 95% CI 0.45–35.79). Controlling for other significant reproductive and lifestyle factors identified in our study subjects did not alter the risk profile. Our analyses was based on the speculation that harboring longer repeat lengths of the CYP19 tetranucleotide repeat polymorphism will increase the risk of breast cancer. This consideration was based on evidence from previous epidemiological studies demonstrating increased risk of breast cancer with longer repeat lengths of the tetranucleotide repeats.

Our findings of increased breast cancer risk in association with harboring longer repeats of the (TTTA)n tetranucleotide repeats have been noted in other populations. Kristensen et al [5], studied the association between the tetranucleotide simple tandem repeat polymorphism and breast cancer risk in a case-control study (sporadic cases: n = 182; familial cases: n = 185; and controls; n = 252) among Norwegian and Swedish individuals and observed a positive association between carriage of the 12 repeat allele [allele [12], (TTTA)_12_] of CYP19 and risk of breast cancer compared with repeat lengths less than 12 (OR = 2.42, 95% CI, 1.03–5.80). These authors also observed a higher frequency of the 12 allele among estrogen- and progesterone receptor positive cases compared with receptor negative cases [5]. Using data from the Nurses' Health Study, Haiman et al [7] examined the relationship between the CYP19 tetranucleotide repeats and breast cancer risk among US Caucasian women; the authors found that harboring the 10 repeat allele [not identified in the Nordic population by Kristensen et al [5]] conferred a 3-fold increased risk of breast cancer (OR = 3.08, 95% CI 1.35–7.01). A non-significant 49% increased risk was also reported by these investigators for individuals harboring the 12 repeat allele (OR = 1.49, 95% CI 0.86–2.56). In a study among Caucasian women in the US, Siegelmann-Danieli and Buetow [6] observed a lower frequency of the 12 allele and higher frequency of the 7 allele among cases (n = 348) than controls (n = 145).

Other studies have not provided support for the association between CYP19 repeat alleles and breast cancer risk. In a multiethnic study [cases/controls: African-American (71/104); Japanese (10/41); Latina (61/99); and non-Latina White (49/66)], Probst-Hensch et al [9], observed equivalent allele (7, 7 (-3 bp), 8, 10, 11, 12, 13) and genotype frequencies among breast cancer cases and controls in all ethnic groups. Likewise, Healey et al [8] observed no relationship between either the 10 or 12 repeat alleles and breast cancer risk among British subjects.

Differences in study size and ethnicity may account for the discrepancy between study results. The studies of Siegelmann-Danieli and Buetow [6] and Probst-Hensch et al [9], had less than 200 Caucasian cases and/or controls, which may result in unreliable allele frequency estimates for rare alleles. The positive association reported by Kristensen et al [5] was observed among Caucasian women of Scandinavian ancestry. High risk CYP19 alleles may be linked to functional genetic variations among specific Caucasian ethnic groups. Consequently, disproportionate frequencies of different Caucasian ancestral groups between cases and controls may influence associations between CYP19 alleles and disease risk.

Because of the weak association detected in most association studies involving single nucleotide polymophisms and breast cancer risk, haplotype-based studies have been proposed as a more powerful comprehensive approach to identify causal genetic variation underlying breast cancer and other complex diseases. Employing these techniques using data from the Multiethnic Cohort (MEC) Study recruiting African-American, Hawaiian, Japanese, Latina and white women, Haiman et al [12] utilized twenty-five haplotype-tagging SNPs (htSNPs) to predict the common haplotypes with high probability within the CYP19 gene and genotyped these htSNPs in a breast cancer case-control study nested in the MEC Study (cases, n = 1355; controls, n = 2580). These investigators observed significant haplotype-effects in block 2 [P = 0.01; haplotypes 2b (OR = 1.23; 95% CI, 1.07–1.40), 2d (OR = 1.28; 95% CI, 1.01–1.62)]. They also found a common long-range haplotype comprising block-specific haplotypes 2b and 3c to be associated with increased risk of breast cancer (haplotype 2b-3c: OR = 1.31; 95% CI, 1.11–1.54). Although this methodology does not require the casual variant to be identified and tested directly, it has the potential to highlight physical regions that harbor putative disease associated variants for further re-sequencing to uncover ethnic-specific disease associated variants.

## Conclusion

Despite the inconsistencies in the epidemiological literature and inconclusiveness of current experimental observations on the role of aromatase in breast carcinogenesis, a combination of experimental [13-17], laboratory [4,18-22] and clinical data [3,23-27] strongly suggests a role for aromatase in the etio-pathological network of breast carcinogenesis. Our finding of a modest increased risk of breast cancer among women harboring 10 or more (TTTA)_n _repeat alleles of the CYP19 (aromatase) gene is in keeping with this proposition. As mentioned previously, some of the current epidemiological studies lack adequate sample size to detect the low risk profile impacted by low-penetrance alleles such as the STRP being considered in this study and this may have affected our results. We hope to further investigate the association between this polymorphism and other variants in the CYP19 gene by recruiting more study participants. There is emerging consensus that future molecular epidemiological studies should focus more on polygenic models employing haplotytpe-based techniques to properly assess the contributions of various low-penetrance genes acting in specific metabolic pathways such as estrogen biosynthesis and metabolism pathway to disease risk. Such studies will require large sample sizes and the collaborative efforts of multi-center investigators. In addition, the combination of high through-put genotyping techniques with emerging technologies including microarray and proteomic technology will enhance our ability to further understand the genetic factors involved in breast carcinogenesis.

## Abbreviations

1. STRP: Simple tandem repeat polymorphism

2. CYP19: Cytochrome P45019A1 gene

3. DNA: Deoxyribonucleic acid

4. PCR: Polymerase chain reaction

5. SNP: Single Nucleotide Polymorphism

6. ABI: Applied Biosystems Incorporated

7. SAS: Statistical Analysis Systems

8. WHR: Waist/hip ratio

## Competing interests

The author(s) declare that they have no competing interests.

## Authors' contributions

MNO, CHB, REF, KLH, participated in conceptualization, design of the study and preparation of manuscript; MNO, ERE, SNCA, JO and EEOU recruited study participants from Nigeria and organized the transfer of biological samples to the University of Pittsburgh; MNO, CHB and JMZ carried out the genetic analysis.
